# Some Thoughts From the New Editor-In-Chief

**DOI:** 10.14309/ctg.0000000000000358

**Published:** 2021-05-06

**Authors:** Brian C. Jacobson

**Affiliations:** 1 Massachusetts General Hospital, Boston, Massachusetts, USA.

It is with great humility that I accept the American College of Gastroenterology Board of Trustees' offer to serve as the new Editor-in-Chief of *Clinical and Translational Gastroenterology* (*CTG*). Following Dr. David C. Whitcomb, MD, PhD, who recently completed an extended term as Editor, is both an honor and a daunting task. I speak for the entire Editorial Board when I thank Dr. Whitcomb for his vision and years of service that saw the launch of the journal and its rise to prominence as a highly regarded publication within our field. Likewise, I want to thank outgoing Associate Editors Max Petrov, Michael Scharl, and Field Willingham for their years of service to *CTG*.

Should I succeed in this new role, it will only be because of the amazing tutelage I received as an Associate Editor at Gastrointestinal Endoscopy under the skillful leadership of then Editor-in-Chief Glenn Eisen. I have such fond memories of serving on that Editorial Board with such luminaries as John Vargo, Klaus Mergener, Marcia Canto, Stuart Sherman, Robert Enns, Jacques Devière, Ananya Das, John Saltzman, Jenifer Lightdale, and Ian Gralnek. That Board had a camaraderie and mission-driven enthusiasm that I hope to recreate at *CTG*. To that end, I am extremely excited to announce the 2021 Editorial Board, including John Kim, David Levinthal, Violeta Popov, Eugenia Shmidt, Elena Stoffel, and Andrew Tai. This is an editor's dream team, and I know they will help continue the *CTG* tradition of excellence.

By way of self-introduction, my career path in Gastroenterology can best be described as nontraditional. I am a general gastroenterologist who also performs interventional endoscopy. My research career has included work on gastroesophageal reflux disease within the Nurses' Health Study and other large cohorts but has also focuses on aspects of colonoscopy including optimization of bowel preparation. I spent 18 years at the Boston University School of Medicine and practiced at Boston Medical Center, the largest safety net hospital in New England, surrounded by incredible colleagues who came to be like family. There, I also served 5 years as the medical director of a new Accountable Care Organization, drawing on prior experiences in quality improvement and population healthcare. Last year, I moved across town to Massachusetts General Hospital to help develop exciting new programs and grow the Gastroenterology Division. Once again, I find myself among wonderful colleagues whose passion for excellence and discovery impress me greatly on a daily basis.

As I assume this new role as Editor-in-Chief, I see an opportunity to reaffirm the journal's focus for its readership and for those authors seeking a platform to share their research findings. As our journal's name makes clear, our purview is both clinical and translational research in the field of gastroenterology. Clinical research is fairly well understood to include patient-oriented research, outcomes research, and health services research. Translational research has, admittedly, a more nuanced definition that has been evolving over decades. The National Institutes of Health (NIH) offers the following description:

*Translational research includes 2 areas of translation. One is the process of applying discoveries generated during research in the laboratory, and in preclinical studies, to the development of trials and studies in humans. The second area of translation concerns research aimed at enhancing the adoption of best practices in the community. Cost-effectiveness of prevention and treatment strategies is also an important part of translational science* (http://grants.nih.gov/grants/guide/rfa-files/RFA-RM-07-007.html; accessed February 7, 2021).

Translational research has been subclassified into 2, 3, and even 5 categories. For authors trying to determine what to submit to *CTG*, it is perhaps easier to define what we do not consider translational research. Scientific discoveries that have not been applied directly to human subjects are better shared elsewhere. For example, the development of potential disease biomarkers may be fundamental to improved health outcomes. However, unless a novel biomarker is being tested among a population with and without disease, we do not consider it translational.

At the other end of the spectrum, we feel the time has come for translational research to include public health dimensions that affect the human experience, including poverty, access to education and healthcare, political unrest, and all forms of inequality.

To truly improve the lives of our patients, we must embrace a scientific approach to understanding and removing barriers to well-being, whatever their form.

We are fortunate in that *CTG* is an open access journal available free of charge to our readers—clinicians, researchers, and public health scientists worldwide. We will capitalize on this by seeking to expand our readership globally, bringing the latest knowledge to those who can apply it immediately to their own research or to make their patients' lives better.

I thank you for making *CTG* a source for your ongoing efforts to further the field of gastroenterology and look forward to reading your research findings over the next several years.

## CONFLICTS OF INTEREST

**Guarantor of the article:** Brian C. Jacobson, MD, MPH.

**Specific author contributions:** B.C.J. wrote and approved the article.

**Financial support:** None reported.

**Potential competing interests:** None reported.

